# Differential Expression of Lipid Metabolism Genes, CROT and ABCG1, in Obese Patients with Comorbid Depressive Disorder and Risk of MASLD

**DOI:** 10.3390/metabo15060392

**Published:** 2025-06-11

**Authors:** Joanna Michalina Jurek, Elena Cristina Rusu, Javier Camaron, Helena Clavero-Mestres, Carmen Aguilar, David Riesco, Belen Xifré, Javier U. Chicote, Salomé Martinez, Marga Vives, Fàtima Sabench, Teresa Auguet

**Affiliations:** 1 Grup de Recerca GEMMAIR (AGAUR)—Medicina Aplicada (URV), Departament de Medicina i Cirurgia, Institut d’Investigació Sanitària Pere Virgili (IISPV), Universitat Rovira i Virgili (URV), Mallafré Guasch 4, 43007 Tarragona, Spain; joannamichalina.jurek@urv.cat (J.M.J.); helena.clavero@urv.cat (H.C.-M.);; 2 Servei Medicina Interna, Hospital Universitari de Tarragona Joan XXIII, Mallafré Guasch 4, 43007 Tarragona, Spain; 3 Servei Medicina Interna, Hospital del Vendrell, Ctra. Barcelona, s/n, El Vendrell, 43700 Tarragona, Spain; 4 Servei Anatomia Patològica, Hospital Universitari de Tarragona Joan XXIII, Mallafré Guasch 4, 43007 Tarragona, Spain; 5 Servei de Cirurgia, Hospital Sant Joan de Reus, Departament de Medicina i Cirurgia, Universitat Rovira i Virgili (URV), Institut d’Investigació Sanitària Pere Virgili (IISPV), Avinguda Doctor Josep Laporte 2, 43204 Reus, Spain

**Keywords:** depression, metabolic dysfunction-associated steatotic liver disease, obesity, lipid metabolism, genetic dysregulation

## Abstract

Background: There is accumulating evidence supporting a bidirectional relationship between metabolic dysfunction-associated steatotic liver disease (MASLD) and depressive disorder (DD), with possible genetic factors related to hepatic lipid metabolism. Our aim was to analyse the prevalence of DD in patients with obesity at risk of MASLD, and to evaluate the hepatic expression of genes involved in lipid metabolism, in patients with DD. Methods: In 152 patients with morbid obesity who underwent bariatric surgery, medical data, blood and liver samples were collected. Liver biopsies were scored for MASLD staging were used for gene expression analysis. Results: The DD prevalence in this cohort was 29.6%, and patients with DD had a significantly higher hepatic expression of the *CROT* and *ABCG1* genes. Moreover, patients in the MASLD group showed significantly higher relative hepatic expression of *SREBP1* and *ABCG1* genes compared to the normal liver group. Some anthropometric and clinical measures (BMI and DBP) were positively correlated with the expression of *SREBP2, ABCG1* and *CROT* genes, while the expression of *CPT1α* was negatively correlated with age, SBP and DBP. There was a positive relationship between GGT and ALP levels and the relative expression of *ABCG1* and *ACC1* genes. Conclusions: In this study, individuals with morbid obesity demonstrated an elevated prevalence of DD. Moreover, hepatic genetic dysregulation of lipid metabolism may influence the interplay between MASLD and DD in patients with morbid obesity.

## 1. Introduction

Obesity is an emerging global chronic condition characterized by excessive body fat accumulation [1], resulting from an imbalance between caloric intake and energy expenditure [2], which affects over 1 billion individuals worldwide [1]. This condition is closely associated with a variety of metabolic and mental disorders, including cardiovascular diseases (CVDs) [1], type 2 diabetes mellitus (T2DM) [1], Metabolic Dysfunction-Associated Steatotic Liver Disease (MASLD) [3] and psychiatric conditions, including depression/depressive disorders (DD) and anxiety [4]. The relationship between obesity and depression is complex and multifactorial [4], driven by shared mechanisms, including combination of psychological, behavioral and biological factors [5]. In particular, metabolic dysregulation resulting from visceral adipose tissue accumulation along with hormonal imbalance, that contributes to increased chronic inflammation and insulin resistance and dyslipdemia [6], may play a crucial role in the progressive development of liver conditions, such as MASLD [3]. Recent research indicates that DD is prevalent among individuals with MASLD, particularly in females, smokers, and those with elevated Body Mass Index (BMI) or T2DM [7]. Obesity is closely related to both depression and MASLD, and having a diagnosis of Depressive Disorder (DD) may additionally influence this relationship and contribute to adverse health outcomes, such as an increased risk of coronary heart disease [8]. In addition, the bidirectional relationship between MASLD and depression is becoming increasingly evident in obese populations. Approximately 14% of individuals with MASLD are diagnosed with clinical depression, and this comorbidity is associated with more severe hepatic histological features, such as hepatocyte ballooning [9]. Notably, increased inflammation and oxidative stress observed in MASLD [10] are also implicated in depression and may impact neurotransmitter metabolism [11]. For example, alterations of gamma-aminobutyric acid (GABA) signaling have been proposed as important genetic modulator of inflammatory cascades [12] in macrophages [13] and epithelial cells, by acting on TLR-4, NF-kb, p65 and MyD88 mRNA expression [14] in MASLD. Although some indications suggest associations between the presence of single nucleotide polymorphisms (SNPs) and increased body mass index (BMI), as well as total and low-density lipoprotein (LDL) cholesterol levels [15], the genetic risk factors related to these psychiatric and metabolic illness need to be further investigated. The observed associations between single nucleotide polymorphisms (SNPs) and increased BMI, as well as alterations in lipid profiles, such as total and low-density lipoprotein (LDL) cholesterol [15], may suggest a role for shared genetic mechanisms linking metabolic dysregulation observed in morbid obesity and MASLD with DD.

Although many candidate genes have been proposed in the management of mental conditions and MASLD [16], those involved in the post-transcriptional regulation of lipid metabolism, cholesterol and fatty acid homeostasis [17,18] seem particularly relevant for individuals with high metabolic risk [19,20]. For example, decreased HDL cholesterol levels have been linked to the presence of an SNP in the *ATP Binding Cassette Subfamily G Member 1* (*ABCA1)* gene in patients with cardiovascular conditions [21], whereas *Carnitine O-Octanoyltransferase* (*CROT*) gene, which is involved in mediating the transfer of fatty acids to mitochondria for β-oxidation, has been proposed as a contributor to MASLD progression, possibly through increased de novo hepatic fat synthesis, inflammation and fibrosis [22].

Similarly, transcription factors such as sterol regulatory element binding proteins (*SREBPs*), including *Sterol Regulatory Element Binding Protein 1c (SREBP1c)* and *Sterol Regulatory Element Binding Protein 2 (SREBP2c*), influence the expression of lipogenic genes [22] and thus affect fatty acid metabolism and cholesterol homeostasis. Inhibition of these factors may ameliorate metabolic dysfunction and liver injury in experimental models of MASLD [23]. Furthermore, the significant role of both congenital genetic alterations and ongoing environmental influences in the pathogenesis of depression [24] is supported by finding that individuals with mental health issues, up to 20 years before diagnosis, had higher levels of glucose, triglycerides, and total cholesterol [25].

In particular, patients with major depressive disorder (MDD) who present with elevated BMI and abnormalities in glucose metabolism appear to exhibit a higher prevalence of lipid metabolism disturbances, specifically, dysregulation such as elevated cholesterol and triglyceride levels [26]. Hyperglycemia, by modulating transcription factors and co-regulators through the protein kinase C and AKT-mTOR signaling pathways, has been shown to promote adipogenesis and contribute to altered lipid profiles [27]. Moreover, emerging evidence suggests that ABC transporter genes, including *ABCB1* and *ABCB6*, influence susceptibility to psychiatric disorders like MDD. For example, polymorphisms in *ABCB1* and *ABCB6* (e.g., rs1109866, rs1109867) have been linked to MDD risk and poorer cognitive performance, suggesting that these genes affect emotional regulation through neurocognitive pathways [28], whereas ABCB1-rs1045642 has been identified as a protective variant for both MDD and bipolar disorder [29].

Exploring the differential expression of hepatic genes involved in lipid metabolism may help elucidate possible mechanisms underlying metabolic dysfunction in DD, especially in patients with elevated BMI who are at risk for MASLD.

Given the possible shared risk factors between obesity, MASLD and MDD, and that one of them appears to be alterations in lipid metabolism, the main aim of this exploratory study is, firstly, to assess the prevalence of DD in a cohort of patients with morbid obesity who underwent bariatric surgery. And, secondly, to explore the differential hepatic expression of genes involved in lipid metabolism based on the presence of DD and, also, according to the presence of MASLD. While the results may not indicate the genetic associations, this study may provide a better understanding of the possible role of hepatic lipid metabolism in individuals with morbid obesity, who report DD and are at risk of MASLD.

## 2. Materials and Methods

### 2.1. Study Population

The study was approved by the institutional review board (Institut d’Investigació Sanitària Pere Virgili CEIm (Comité Ético de Investigación con medicamentos, Drug Research Ethics Committee in English): 23c/2015), and all participants provided written informed consent.

In this study, a total of 152 patients with morbid obesity who underwent bariatric surgery were recruited, including 15 men (9.9%) and 137 women (90.1%). The exclusion criteria, biochemical analysis, and MASLD diagnoses were previously described [20]. Briefly, in those patients with suspected liver disease, hepatic biopsies were obtained during planned laparoscopic bariatric surgery. All liver biopsies were performed for clinical diagnosis purposes. The exclusion criteria were as follows: (1) individuals who had alcohol consumption higher than 10 g/d; (2) patients who had acute or chronic hepatic, inflammatory, infectious, or neoplastic diseases; (3) menopausal women or women using contraceptives to avoid the interference of hormones that can cause biases in glucose and lipid metabolism; (4) patients with T2DM receiving pioglitazone; and (5) patients treated with antibiotics in the previous 4 weeks. Patients’ clinical histories, along with all other variables, included information on whether they had been diagnosed with a depressive syndrome and/or had received antidepressant treatment. According to this, patients were classified as either patients with DD or patients without depressive disorder (control group, CN).

### 2.2. Hepatopathological Diagnosis

Liver samples were scored by experienced hepatopathologists using the methods described elsewhere [30,31], including hematoxylin-eosin and Masson’s trichrome stains. Briefly, hepatic pathology was considered as simple steatosis (SS) if more than 5% but less than 33% of hepatocytes affected (grade 1—mild SS); then if 33% to 66% of hepatocytes affected (grade 2—moderate SS); and finally, if more than 66% of hepatocytes affected (or grade 3—severe SS). In addition, hepatic tissue was assessed based on the minimum criteria for the diagnosis of steatohepatitis, which included the presence of lobular inflammation and either ballooning cells or perisinusoidal/pericellular fibrosis in zone 3 of the hepatic acinus.

### 2.3. Anthropometric Evaluation and Biochemical Analysis

The anthropometric evaluation and biochemical analysis carried out have been previously described [20]. Briefly, all participants included in the study underwent physical, anthropometric, and biochemical evaluations. Blood samples were extracted using a BD Vacutainer^®^ system (BD IBERIA S.L., Madrid, Spain) by trained hospital nurses after overnight fasting and before surgery. Venous blood samples were obtained in tubes with or without ethylenediaminetetraacetic acid (EDTA), and separated into plasma and serum aliquots by centrifugation (1507 relative centrifugal force, 4 °C, 15 min). A conventional automated analyser (Atellica Systems Analyser, Siemens Healthineers, Erlangen, Germany) was used for the biochemical assessment. Insulin resistance was estimated using the homeostatic model assessment 1 for insulin resistance (HOMA1-IR).

### 2.4. RNA Isolation and Analysis of the Gene Expression in Liver and Serum Samples

The procedure of RNA isolation and quantification was previously described [19]. Briefly, hepatic samples obtained during bariatric surgery were immediately preserved in RNAlater (Sigma, Barcelona, Spain) for 24 h at 4 °C and then stored at −80 °C. TaqMan Assays predesigned by Applied Biosystems (Foster City, CA, USA) were used to detect genes of interest, including *Sterol Regulatory Element Binding Protein 1c (SREBP1c)* and *Sterol Regulatory Element Binding Protein 2 (SREBP2c), Acetyl-CoA Carboxylase 1 (ACC1), Carnitine Palmitoyltransferase 1 Alpha* (*CPT1α), CROT*, *ATP Binding Cassette Subfamily A Member 1* (*ABCA1)* and *ATP Binding Cassette Subfamily G Member 1 (ABCG1);* and 18S ribosomal RNA serving as a housekeeping gene. The total RNA was isolated in accordance with the manufacturers’ protocols RNeasy Mini kit (Qiagen, Barcelona, Spain). cDNA was synthesized using a High Capacity RNA-to-cDNA Kit (Applied Biosystems). The specific primers for each selected genes were used, as follows *SREBP1c* (HS01088691_M1), *SREBP2c* (Hs01081784_m1), *ACC1* (HS00167385_M1), *CPT1α* (Hs00912671_m1), *CROT* (Hs00221733_m1), *ABCA1* (Hs01059118_m1) and *ABCG1* (Hs00245154_m1), all supplied from Applied Biosystems. All reactions were carried out in duplicate in 96-well plates using the 7900HT Fast Real-Time PCR systems (Applied Biosystems).

### 2.5. Statistical Analysis

Data analyses were performed by using the SPSS software for Mac statistical package (version 27.0; SPSS, Chicago, IL, USA). Outliers were removed prior to any statistical analysis, using the interquartile range (IQR) method, to minimize bias in the results. The distribution of variables was obtained using the Kolmogorov–Smirnov test. All results are expressed as the median and the interquartile range (25th–75th). The anthropometric and biochemical variables were compared by independent *t*-test. The different comparative analyses were performed using a nonparametric Mann–Whitney U test for all study groups. Comparisons between groups for categorical variables were performed using cross-tabulations with the chi-square test. The strength of association between variables was calculated using Spearman’s pairwise correlations. Additionally, binary logistic regression models adjusted for age and sex were used to assess the association between depressive disorder and comorbid conditions. *p* values < 0.05 were considered statistically significant.

## 3. Results

### 3.1. Characteristics of Study Participants

In this study, a total of 152 patients with morbid obesity undergoing bariatric surgery were recruited. The cohort included 15 men (9.9%) and 137 women (90.1%). According to their medical history and reported medication use, both men and women were divided into two groups: a DD group consisting of patients who had a depression diagnosis and/or received treatment with anti-depressants (DD group, *n* = 45); and those who had no depression and had not taken antidepressants (CN, *n* = 107). The prevalence of DD in this cohort was 29.6%. The clinical characteristics and biochemical parameters of the cohort are presented in Table 1. The groups were compared in terms of general anthropometric and clinical parameters, including height, BMI, systolic (SBP) and diastolic (DBP) blood pressure; as well as indicators of metabolic status (e.g., HOMA index, glycosylated haemoglobin (HbA1c), glucose and insulin blood levels), lipid profile (e.g., cholesterol, high-density lipoprotein cholesterol (HDL-C), low-density lipoprotein cholesterol (LDL-C), and triglycerides) and hepatic and inflammation biomarkers (e.g., AST, ALT, GGT, and alkaline phosphatase (ALP)) and inflammation (C-reactive protein (CRP)).

The results of comparisons between groups demonstrated no significant differences between participants in the CN and DD groups, except for age and gender (Table 1).

Table 2 shows the medications taken by the patients included in the study for various pathologies, along with a comparison between those with DD or those without. The comparison between the CN and DD groups has shown that DD patients had significantly higher intakes of antidepressants, benzodiazepines and antiepileptics (*p* < 0.05). No significant differences were observed between the groups in the intake of other medications or vitamin supplements (Table 2).

### 3.2. Liver Histology Assessment According to Depression Diagnosis

The hepatic histopathology results were used to classify and determine the incidence of liver pathology in relation to DD. Liver samples from all study participants were assessed histopathologically and classified according to Brunt’s criteria. The comparative analysis of the occurrence and type of hepatic pathology is presented in Table 3. Briefly, there were no statistically significant differences between CN and DD groups, either in terms of hepatic diagnosis or liver histology (*p* < 0.05). There were no additional differences in other forms of liver pathology reported in the participants in this cohort. Moreover, no differences were found in the presence of MASLD or liver histopathology diagnosis when the cohort was subdivided in patients receiving antidepressant treatment and those bit receiving it.

### 3.3. Association Between Depressive Disorder and Comorbid Conditions

To better account for potential confounding factors, we assessed the association between depressive disorder and common metabolic comorbidities using binary logistic regression models adjusted for age and sex. Table 4 presents the odds ratios and *p*-values for each comorbid condition. No statistically significant associations were observed between depressive disorder and the presence of T2DM, Metabolic Syndrome, High Blood Pressure, or MASLD.

### 3.4. Evaluation of the Hepatic Gene Expression Between Cohorts

#### 3.4.1. Evaluation of the Hepatic Gene Expression Between CN and DD Cohorts

**Figure 1:**
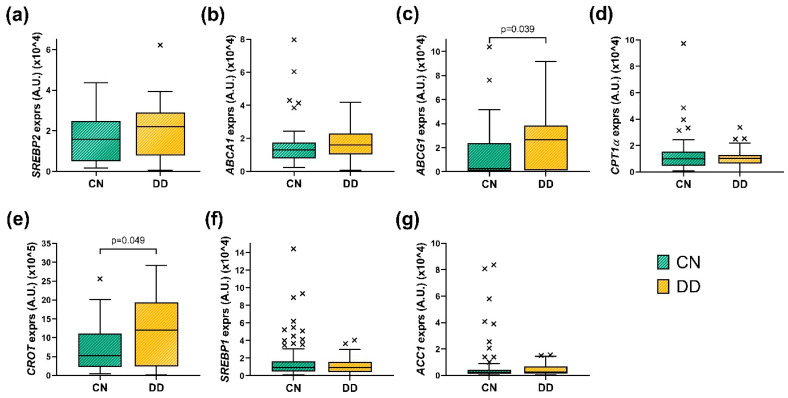
Boxplots presenting the hepatic levels of relative expression of (**a**) *SREBP2*, (**b**) *ABCA1*, (**c**) *ABCG1*, (**d**) *CPT1α*, (**e**) *CROT*, (**f**) *SREBP1*, and (**g**) *ACC* in CN and DD groups. Each boxplot displays the median and interquartile range (IQR). The *x* marks indicate outliers, which were identified using the IQR method. *SREBP*, Sterol Regulatory Element-Binding Protein; *ABCA1*, ATP-Binding Cassette Transporter A1; *ABCG1*, ATP-Binding Cassette Subfamily G Member 1; *CROT*, Carnitine O-Octanoyltransferase; *CPT1α*, Carnitine Palmitoyltransferase 1α; *ACC*, Acetyl-CoA Carboxylase; DD, Depression Disorder Group; CN, control group. A.U, arbitrary units. Mann–Whitney test was used to calculate the difference between groups. Significant *p* values are annotated (*p* < 0.05).

The second objective of this study was to examine the expression of genes involved in hepatic lipid metabolism in liver biopsy samples from the CN and DD groups (Figure 1). Briefly, individuals in the DD group had significantly higher relative hepatic expression of *CROT* and *ABCG1* genes compared to those in the CN group (*p* < 0.05). There were no other differences observed in the relative expression of the remaining genes studied in the liver biopsy samples.

#### 3.4.2. Evaluation of the Hepatic Gene Expression in Respect to Liver Histology

**Figure 2:**
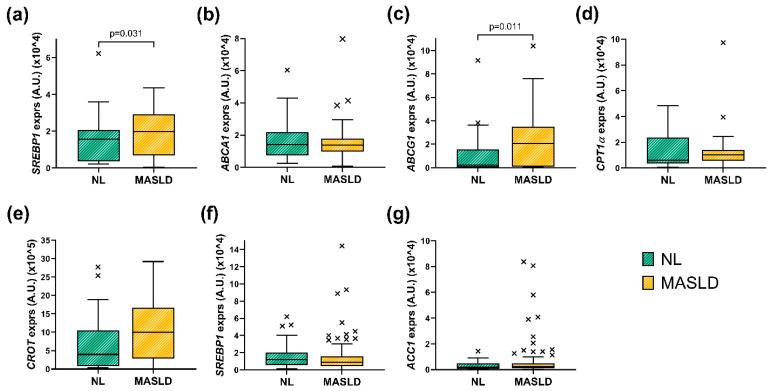
Boxplots presenting the hepatic levels of relative expression of (**a**) *SREBP2*, (**b**) *ABCA1*, (**c**) *ABCG1*, (**d**) *CPT1α*, (**e**) *CROT*, (**f**) *SREBP1*, and (**g**) *ACC* in NL and MASLD groups. Each boxplot displays the median and interquartile range (IQR). The *x* marks indicate outliers, which were identified using the IQR method. *SREBP*, Sterol Regulatory Element-Binding Protein; *ABCA1*, ATP-Binding Cassette Transporter A1; *ABCG1*, ATP-Binding Cassette Subfamily G Member 1; *CROT*, Carnitine O-Octanoyltransferase; *CPT1α*, Carnitine Palmitoyltransferase 1α; *ACC*, Acetyl-CoA Carboxylase; NL, Normal Liver; MASLD, metabolic dysfunction-associated steatotic liver disease. A.U, arbitrary units. Mann–Whitney test was used to calculate the difference between groups. Significant *p* values are annotated (*p* < 0.05).

Accordingly, we analysed the expression of the genes involved in hepatic lipid metabolism in liver biopsy samples according to hepatic histological diagnosis. This approach enabled us to distinguish between patients with MASLD (MASLD group) and those with normal liver histology (NL group) (Figure 2). Briefly, patients in the MASLD group showed significantly higher relative hepatic expression of *SREBP1* (*p* = 0.031) and *ABCG1* (*p* = 0.011) genes compared to the NL group. No significant differences were observed in the expression of the other genes analysed.

### 3.5. Correlations Between the Expression of Hepatic Genes Involved in the Lipid Metabolism with Clinical and Biochemical Measures of the Cohort

**Figure 3:**
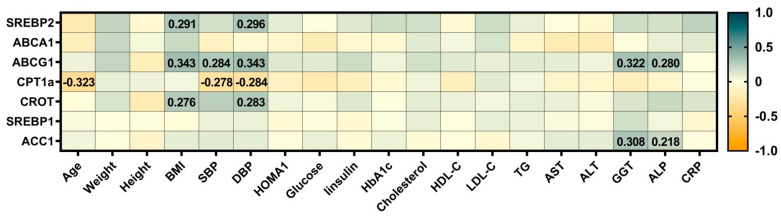
Heat-map presenting the relationship between the expressions of hepatic genes involved in lipid metabolism and the anthropometric measures, hepatic and immune-metabolic indicators measured in the total cohort. *SREBP*, Sterol Regulatory Element-Binding Protein; *ABCA1*, ATP-Binding Cassette Transporter A1; *ABCG1*, ATP-Binding Cassette Subfamily G Member 1; *CROT*, Carnitine O-Octanoyltransferase; *CPT1α*, Carnitine Palmitoyltransferase 1α; *ACC*, Acetyl-CoA Carboxylase; BMI, body mass index; SBP, systolic blood pressure; DBP, diastolic blood pressure; HOMA1-IR, homeostatic model assessment method–insulin resistance; HbA1c, glycosylated hemoglobin; TG, triglycerides; HDL-C, high-density lipoprotein cholesterol; LDL-C, low-density lipoprotein cholesterol; AST, aspartate aminotransferase; ALT, alanine aminotransferase; GGT, gamma-glutamyltransferase; ALP, alkaline phosphatase; CRP, C-reactive protein. Only significant Spearman’s ρ are displayed. *p* < 0.05 were considered significant.

To assess the relationship between the expression of hepatic genes involved in lipid metabolism and clinical and biochemical parameters in this cohort, a correlation analysis was performed, and the results are presented in Figure 3.

The results showed the presence of correlations between expression of hepatic genes implicated in lipid metabolism and some anthropometric, hepatic and metabolic outcomes, also including biomarkers of liver function, GGT and ALP, which were positively correlated with the expression of *ABCG1* and *ACC1* genes.

## 4. Discussion

There is growing evidence to suggest a bidirectional link between depression and metabolic complications such as dyslipidemia [6], arising from obesity [4,32], including MASLD [3], with a proposed role of perturbed lipid metabolism [15,33,34,35]. Therefore, the main purpose of this study was to assess the prevalence of DD in a cohort of patients with morbid obesity and at risk for MASLD, and also to analyse the differential hepatic expression of genes related to lipid metabolism in relation to their reported DD symptoms and the presence of MASLD. The main results of this research demonstrated that patients with DD had significantly increased hepatic expression of the *CROT* and *ABCG1* genes compared to patients without DD. Moreover, patients in the MASLD group showed significantly higher hepatic expression of the *SREBP1* and *ABCG1* genes compared to the NL group. Furthermore, anthropometric parameters, including BMI and DBP, were positively correlated with expression of *SREBP2*, *ABCG1* and *CROT* genes, while the expression of *CPT1α* was negatively correlated with age, SBP and DBP measures. Liver enzymes, GGT and ALP, were positively associated with the expression of *ABCG1* and *ACC1* genes.

Regarding the prevalence of DD in this cohort, it was 29.6%, which is similar to the ranges reported previously in other studies conducted on individuals with liver conditions [7,33], including MASLD [36]. However, this prevalence was lower than that reported in obese patients undergoing bariatric surgery (48.2%) [37], as well as in other cohort studies [38,39,40], such as 12.3% in the UK [39] and 20.79% in China [40]. No significant differences were observed between the DD and CN groups in this cohort for anthropometric measures or indicators of metabolic health, including glycaemic control, lipid profiles and hepatic status. Moreover, we did not detect differences between the DD and CN groups in relation to liver histopathology. Therefore, in our cohort, we were not able to reproduce the hypothesis that the presence of depression diagnosis, along with metabolic disturbances, could potentially influence MASLD progression, or that psychiatric disorders increase the risk and severity of MASLD and its complications, such as severe hepatocyte ballooning [9,35] and hepatic steatosis [33,41]. The fact that this is a morbidly obese population could potentially mask this relationship.

Evidence supporting the role of differential expression of hepatic genes in the development of psychiatric conditions is limited to studies suggesting that chronic inflammation present in MASLD patients [36] may affect the nervous system and potentially lead to the development of depressive symptoms [34,42]. It has been suggested that increased systemic inflammation may impact neuroendocrine responses (e.g., activation of the hypothalamic-pituitary-adrenal axis) [42] and affect neurotransmitter signalling in the brain [11]. On the other hand, treatment with some antidepressant drugs may additionally influence cardiometabolic risk [43] or even lead to hepatotoxicity [44], while others, such as selective serotonin reuptake inhibitors and selective noradrenaline reuptake inhibitors, may offer safer therapeutic options, especially for patients with chronic liver disease and those who have undergone liver transplants [45]. However, in our study, the presence of MASLD or more severe histopathology was not found to be increased in patients with this disease who were receiving antidepressant treatment. Unfortunately, we did not analyse the length of time the patients were subjected to this treatment, due to lack of information.

Although there is growing evidence indicating that alterations in the hepatic metabolism may be related to the occurrence of depression [42,46,47], more studies are needed to characterize this interaction. Our pilot study, for the first time, using a comparative analysis stratified by the presence of DD, demonstrated a significant difference in the hepatic expression levels of the *CROT* and *ABGC1* genes. Furthermore, when we analysed these genes after stratifying participants by the histology assessment, we demonstrated that those with confirmed MASLD diagnosis had significantly higher relative hepatic expression of *SREBP1* and *ABCG1* genes compared to the NL group, like it was observed in our previous studies [19,20].

All together, these early findings may indicate a possible role of shared molecular mechanism involved in lipid metabolism and the pathology of both MASLD and depression in subjects with comorbid obesity.

The role of *CROT* in lipid metabolism and the beta-oxidation of fatty acids is well-established [48], and genetic variants of the enzyme located in the carnitine shuttle-*CROT* have been linked to coronary artery disease [49]. In addition, the reported association between the rs2214930-CC polymorphism in the *CROT* gene and decreased HDL-C levels [49] may indicate its potential role in determining the risk of metabolic conditions [49]. Interestingly, a genetic *CROT* deletion has been linked with increased levels of anti-inflammatory bioactives, including dicarboxylic acids, tetradecanedioic acid and azelaic acid in the liver and plasma of dyslipidemic LDL receptor-deficient mice, also followed by an increase in omega-3 polyunsaturated fatty acid, especially eicosapentaenoic acid (EPA) [50]. Therefore, these findings may suggest the relevance of *CROT* gene, as potential modulator involved in lipid homestatsis, thus reinforcing the gene contribution to the metabolic and psychiatric disturbances observed in MASLD patients with reported DD.

In relation to the *ABCG1* gene, being involved in the transport of lipids, including cholesterol, phospholipids, sphingomyelin and oxysterols, it significantly contributes to the maintenance of lipid homeostasis and may be considered a risk factor for cardiometabolic health [51]. *ABCG1,* as a Liver-X-receptors (LXRs) -responsive gene, was proposed in regulating the sterol biosynthetic pathway; thus, excess *ABCG1* was associated with decreased levels of sterol precursors and increased levels of *SREBP2* in experimental murine models [52]. This early evidence from the experimental studies may suggest a presence of the feedback loop between ABC gene family and their regulators, SREBP, which may participate in lipid metabolism modulation, thereby influencing a cellular lipid content.

Interestingly, the expression of one of the *ABCB1* gene products, a p-glycoprotein, has been intensively studied in the clinical efficacy of treatment with antidepressants, whereas *ABCB1* polymorphisms have been used as possible explanation of the individual differences in the response to these medications [53]. Beyond their established role in drug transport, *ABCB1* is becoming recognized for its involvement in neuropsychiatric conditions, as the most recent evidence suggests a broader role for this gene and its polymorphisms in MDD. For instance, specific variants in *ABCB1* and *ABCB6* (e.g., *rs1109866*, *rs1109867*) were significantly associated with MDD risk and performance on cognitive tests, overall suggesting that ABC transporter gene variants may influence depression severity through pathways involving executive function and cognitive flexibility [28]. In addition, the ABCB1-rs1045642 T allele has been recently proposed as a potential protective factor for mental disorders, including both MDD and bipolar disorder, which further supports the relevance of investigating the members of ABC family members, in determining psychiatric risk in individuals with co-occurring cardiometabolic and mental disorders [28]. Interestingly, the activity of central regulators of lipid homeostasis, SREBPs, has been implicated in the development of MASLD [29], as it can be triggered by excessive caloric intake, insulin resistance, or endoplasmic reticulum stress, contributes to the development of metabolic syndrome and MASLD [54]. Furthermore, overexpression of SREBP-1c has been shown to increase de novo lipogenesis, hepatic lipid accumulation, and insulin resistance, which could affect the pathogenesis of MASLD [55]. This mechanism could also be relevant to individuals with DD, especially those taking antipsychotics, as certain antidepressants have been shown to trigger activation of the SREBP system and, subsequently, lipogenesis-related genes to varying degrees (depending on the drug used) in in vitro studies [56]. Taken together, these observations may provide an early evidence supporting the role of genetic alterations in the lipid metabolism pathways including ABC transporters and their central regulators in the MASLD pathogenesis and DD symptoms presence. Also, further studies focusing on the bidirectional relationship between these conditions could provide a biological explanation reported in this study.

Although these observations are promising, especially in the context of preserving fatty acid metabolism and preventing mitochondrial dysfunction, more studies need to be conducted. Furthermore, dysregulation of lipid metabolism has also been associated with depression [57], showing that individuals diagnosed with MDD with a long symptom duration, equal or greater than 3 years, had lower levels of HDL-C compared with healthy controls or MDD patients with shorter symptom duration [57]. Interestingly, the probability for long symptom duration doubled for each 0.5-mmol/L decrease in HDL-C levels when accounting for confounding factors [57], whereas male subjects had a 1.041-fold higher probability of depression diagnosis for a 1-unit increase in TG/HDL-C ratio [58].

Further analysis focused on the correlations between hepatic gene expressions related to lipid metabolism, immuno-metabolic biomarkers and anthropometric measures and identified a series of significant associations between the expressions of genes implicated in lipid metabolism, such as *SREBP2*, *ABCA1*, *ABCG1*, and *CROT*, and anthropometric measures, including age, BMI, DBP and SBP, as well as biomarkers of liver function, GGT and ALP, which consistently have been shown in our previous studies [19,20].

A key advantage of this study is that it includes a well-characterized cohort of patients with morbid obesity at MASLD risk. The exploratory approach of this study significantly contributes to understanding the potential role that genes related to hepatic lipid metabolism play in relation to DD in patients with morbid obesity. Nevertheless, the study has some limitations. The initial cohort was not specifically designed to evaluate psychiatric health outcomes, and the classification of DD was based on clinical history, as determined by the doctor during the examination and on antidepressant treatment. Therefore, we do not have data from a validated depression scale or a comprehensive psychiatric evaluation, which may limit diagnostic rigor. In addition, due to the lack of a non-obese population in this study, we cannot confirm that the relationship between MASLD and DD might simply reflect the known pathways through which obesity impacts both the liver and mental health, as well as whether certain alterations in liver biology could be related to the treatment with certain drugs, including antidepressants. It is also important to note that the results of this pilot study might be influenced by biases between DD and CN groups as well as gender, as the majority of participants were women (70% of patients who undergo bariatric surgery in our center are female). Further studies including more patients and of both sexes are needed to corroborate our results.

Given these factors, the results of this exploratory study require follow-up validation based on comprehensive psychiatric evaluations and the use of validated depression and anxiety scales. While the dysregulation of genes such as *CROT* and *ABCG1* may play a role in the interplay between MASLD and DD, it is important to recognize that these observations may not be sufficient to fully acknowledge the complex interplay between other metabolic and psychological factors involved influencing the MASLD and DD risk. Consequently, these findings cannot be extrapolated to non-obese MASLD cohorts and should be interpreted with caution, and treated as initial effort into better understanding the modifiers of psychometabolic health.

## 5. Conclusions

In conclusion, the results of this pilot study identify, for the first time, significant associations between genetic dysregulation of hepatic lipid metabolism involving the *CROT* and *ABCG1* genes and the presence of DD symptoms in patients with morbid obesity and implicated MASLD risk. Additionally, patients with confirmed MASLD had significantly higher hepatic expression of *SREBP1* and *ABCG1* genes compared to the NL group. Although these findings are preliminary, they may suggest a potential link between altered lipid metabolic pathways and the presence of MASLD and DD. This early-stage hypothesis requires further validation in larger, well-characterized cohorts to improve understanding of the shared pathophysiology of MASLD and DD in the context of metabolic disturbances. However, these findings underline the importance of a comprehensive psychological assessment in patients with morbid obesity and/or MASLD and suggest that interventions aimed at improving metabolic health could have a positive impact on patients’ mental health.

## Figures and Tables

**Table 1:** Anthropometric and biochemical variables in the cohort in respect to the study groups.

Variable	Total Cohort (*n* = 152)	CN Group (*n* = 107)	DD Group (*n* = 45)	*p* Value
Age (years)	49.0 (39.8–56.1)	48.6 (39.3–55.1)	50.4 (42.9–58.9)	0.035 *
Gender (*n*, % females)	137 (90.1%)	92 (86.0%)	45 (100%)	0.008 *
Weight (kg)	119.0 (108.0–131.8)	120.0 (107.7–132.0)	117.0 (109.0–128.5)	0.884
Height (m)	1.6 (1.6–1.7)	1.6 (1.6–1.7)	1.6 (1.6–1.6)	0.054
BMI (kg/m^2^)	46.2 (43.1–49.8)	45.9 (42.7–48.9)	47.0 (43.9–51.6)	0.064
SBP (mmHg)	133.0 (120.0–145.0)	133.0 (120.0–142.5)	137.0 (120.0–150.0)	0.455
DBP (mmHg)	75.0 (65.0–85.0)	71.0 (65.0–85.0)	80.0 (63.0–89.0)	0.319
HOMA-1R	4.8 (2.5–8.2)	5.0 (2.6–8.2)	4.0 (1.6–7.5)	0.737
Glucose (mg/dL)	107.0 (89.0–133.0)	105.0 (89.8–133.5)	112.0 (87.5–134.0)	0.955
Insulin (mUI/L)	16.0 (8.6–24.8)	16.0 (8.5–24.9)	15.3 (8.8–24.2)	0.798
HbA1c (%)	5.5 (5.0–6.1)	5.5 (5.0–6.1)	5.6 (5.1–6.7)	0.251
Cholesterol (mg/dL)	168.6 (148.0–195.3)	169.5 (149.5–192.6)	166.9 (147.0–200.9)	0.823
HDL-C (mg/dL)	38.0 (33.0–44.0)	38.0 (33.0–43.8)	38.3 (31.3–47.0)	0.509
LDL-C (mg/dL)	98.0 (80.0–122.6)	97.0 (82.5–123.6)	100.1 (78.0–118.8)	0.920
TG (mg/dL)	151.0 (117.0–205.8)	153.0 (116.5–206.8)	147.5 (117.0–203.0)	0.970
AST (UI/L)	34.0 (24.0–48.8)	33.0 (24.6–46.0)	34.0 (21.0–56.0)	0.903
ALT (UI/L)	35.0 (24.8–53.0)	35.0 (25.5–53.0)	35.0 (24.0–54.5)	0.914
GGT (UI/L)	24.0 (14.5–45.5)	21.6 (14.0–42.3)	33.0 (17.5–52.2)	0.071
ALP (Ul/L)	69.0 (56.0–77.5)	68.0 (53.5–78.0)	69.0 (57.0–76.5)	0.562
CRP (mg/dL)	10 (0.5–2.0)	1.0 (0.5–2.0)	1.0 (0.4–2.0)	0.537

CN, control group; DD, Depression disorder group; BMI, body mass index; SBP, systolic blood pressure; DBP, diastolic blood pressure; HOMA-1R, homeostatic model assessment method–insulin resistance; HbA1c, glycosylated hemoglobin; TG, triglycerides; HDL-C, high-density lipoprotein cholesterol; LDL-C, low-density lipoprotein cholesterol; AST, aspartate aminotransferase; ALT, alanine aminotransferase; GGT, gamma-glutamyltransferase; ALP, alkaline phosphatase; CRP, C-reactive protein. Data are expressed as the median (interquartile range) except gender, which is displayed as percentage (%) of females. *p* values were presented as unadjusted. * Significant differences between CN and DD group (*p* < 0.05).

**Table 2:** Comparison of the reported medication use between the CN and DD groups.

Medication	CN Group (*n* = 107)	DD Group (*n* = 45)	Xi2 (df)	*p* Value
Antidepressants	0 (0%)	39 (86.7%)	123.856 (1)	<0.001 *
Antihypertensive	49 (45.8%)	27 (60%)	2.557 (1)	0.110
Lipid-lowering agents—Statins	17 (15.9%)	13 (28.9%)	3.380 (1)	0.066
Lipid-lowering agents—Fibrates	4 (3.7%)	2 (4.4%)	0.042 (1)	0.838
Diabetes treatment—Insulin	5 (4.7%)	3 (6.7%)	0.253 (1)	0.615
Diabetes treatment—Oral	25 (23.4%)	14 (31.1%)	0.997 (1)	0.318
Analgesics	10 (9.4%)	4 (8.9%)	0.011 (1)	0.916
Analgesics—opioids	3 (2.8%)	1 (2.2%)	0.045 (1)	0.831
Anticoagulants—oral	1 (0.9%)	1 (2.2%)	0.395 (1)	0.530
Antiepileptics	0 (0%)	4 (8.9%)	9.679 (1)	0.002 *
Antihistamines	2 (1.9%)	0 (0%)	0.860 (1)	0.354
Anti-inflammatories	8 (7.5%)	8 (17.8%)	3.490 (1)	0.062
Benzodiazepines	5 (4.7%)	18 (40%)	30.457 (1)	<0.001 *
Corticosteroids	1 (0.9%)	1 (2.2%)	0.395 (1)	0.530
Morphine	0 (0%)	1 (2.2%)	2.371 (1)	0.124
Hepatoprotectors	1 (1%)	0 (0%)	0.422 (1)	0.516
Vitamins	5 (4.9%)	4 (8.9%)	0.864 (1)	0.353

CN, control group; DD, Depression disorder group; df, degree of freedom. Data presented as number of subjects with the condition (percentage of subjects in the corresponding study group). Comparisons between groups were made by cross-tabs with chi-square test. *p* values were presented as unadjusted. * Significant differences between CN and DD group (*p* < 0.05).

**Table 3:** Comparison of the hepatic diagnosis and liver histology assessment between the CN and DD groups.

Hepatic Diagnosis	CN Group (*n* = 107)	DD Group (*n* = 45)	Xi2 (df)	*p* Value
NL	18 (16.8%)	10 (22.2%)	3.429 (2)	0.180
SS	31 (29%)	18 (40%)		
MASH	58 (54.2%)	17 (37.8%)		
Liver histology				
Steatosis	89 (83.2%)	35 (77.8%)	0.615 (1)	0.433
Lobular inflammation	16 (55.2%)	6 (40%)	0.910 (1)	0.340
Ballooning	16 (55.2%)	6 (40%)	0.910 (1)	0.340
Liver fibrosis	12 (11.2%)	4 (8.9%)	0.182 (1)	0.670

NL, normal liver; SS, simple steatosis; MASH, metabolic dysfunction-associated steatohepatitis; df, degree of freedom. Data presented as number of subjects with the condition (percentage of subjects in the corresponding study group). Comparisons between groups were made by cross-tabs with chi-square test. *p* value is presented as unadjusted.

**Table 4:** Association between DD and comorbid conditions based on logistic regression models.

Comorbid Condition	CN Group (*n* = 107)	DD Group (*n* = 45)	OR	*p* Value
T2DM	32 (30.5%)	15 (33.3%)	0.943	0.883
Metabolic Syndrome	83 (77.6%)	33 (73.3%)	0.928	0.928
High Blood Pressure	49 (55.1%)	23 (57.5%)	1.189	0.672
MASLD	89 (83.2%)	35 (77.8%)	0.851	0.720

CN, control group; DD, Depressive disorder group; OR, odds ratio; T2DM, Type 2 Diabetes Mellitus; MASLD, Metabolic dysfunction-associated steatotic liver disease. Data presented as number of subjects with the condition (percentage of subjects in the corresponding study group). Odds ratios refer to the association between depressive disorder and each comorbidity, adjusted for age and sex, based on binary logistic regression models.

## Data Availability

The data supporting the findings of this study are available within the publication and upon reasonable request to the corresponding author.
